# The epidemiological profile of meningitis among adults in a South African district hospital

**DOI:** 10.11604/pamj.2022.41.256.30015

**Published:** 2022-03-29

**Authors:** Kian Terwin, Mari Ferreira, Christiaan Minnie, Kalliopy Marangellis, Ruben Darby, Jesse Berlyn, Anél Kleingeld, Shezree Tiel, Matthew Olukayode Abiodun Benedict, Cornel van Rooyen, Anthonio Oladele Adefuye, Joseph Bukulu Sempa

**Affiliations:** 1 Undergraduate Medical Programme, School of Clinical Medicine, Faculty of Health Sciences, University of the Free State, PO Box 339, Bloemfontein, 9300, South Africa,; 2 Department of Family, Medicine Faculty of Health Sciences, University of the Free State, PO Box 339, Bloemfontein, 9300, South Africa,; 3 Department of Biostatistics, University of the Free State, PO Box 339, Bloemfontein, 9300, South Africa,; 4 Division of Health Sciences Education, Office of the Dean, Faculty of Health Sciences, University of the Free State, PO Box 339, Bloemfontein, 9300, South Africa

**Keywords:** Meningitis, national district hospital, adult, Free State

## Abstract

**Introduction:**

understanding the epidemiological profile of a disease in a particular region allows for proper planning of public health resources for prevention, early diagnosis and treatment. In this present study, we describe the epidemiological profile of viral, fungal, tuberculous and bacterial meningitis among adults at National District Hospital (NDH), Free State province, over three years period (January 2017 to December 2019).

**Methods:**

a retrospective, observational study of all adult meningitis cases, managed at the National District Hospital (NDH) Bloemfontein, Free State Province, South Africa between January 2017 and December 2019.

**Results:**

of the 236 case files reviewed, majority (93.2%; n=220) of the patients managed for meningitis were black, as well as males (55.5%; n = 131). Higher incidence was found between the ages 20 to 49 (81.7%). Of those who died, the majority (n = 14; 63.6%) were males, in the age group 40-49 (n = 7; 31.8%), had TB meningitis (n = 12; 54.5%), were HIV positive (n = 20; 90.9%), and had cell count <100 cells/mm^3^ (n = 10; 45.5%).

**Conclusion:**

our study suggests that combining information on patient demography, co-morbidities, clinical presentation, and examination findings can substantially contribute to raising clinical suspicion, leading to swift identification, diagnosis, and treatment of patients.

## Introduction

Despite the development of numerous potent antimicrobial, introduction of vaccine initiatives and improved critical care, meningitis remains a debilitating illness of serious public health concern, globally [1]. The disease can be caused by many different pathogens including bacteria, fungi, viruses or parasite and in some cases; non-infectious agents could cause it as seen in cancers, systemic lupus erythematosus (lupus) and head injuries [2]. The highest global burden is seen with bacterial meningitis [1]. In many parts of the world, disease occurrence is predominantly sporadic with seasonal variation [1]. However, regular epidemics are observed in the “meningitis belt”, a region that stretches from Senegal (West Africa) to Ethiopia (Eastern Africa) in sub-Saharan Africa [3,4]. In South Africa (SA), the annual incidence rate of meningitis is estimated to be 4 per 100,000 cases in the general population, and occurrence is commonly seen in infants (with an incidence of 40 per 100,000) [5]. Nonetheless, the high dual burden of HIV infection and tuberculosis (TB) has led to changes in the spectrum of causes and outcome of meningitis among adults in SA [6].

The occurrence of infectious diseases vary by geographic region and population, and they may change over time. Wilson (2010) reports that delivering adequate and effective patients care requires an understanding of the basic epidemiological factors that underlie the geography of human diseases and events that cause shifts in the distribution and burden of specific diseases [7]. Furthermore, understanding the epidemiological profile of a disease in a particular region allows for proper planning of public health resources for prevention, early diagnosis and treatment [6]. In this present study, we describe the epidemiological profile of viral, fungal, tuberculous and bacterial meningitis among adults at National District Hospital (NDH), Free State province, over a period of three years (January 2017 to December 2019). In addition, we compared trends in incidence and proportions as well as seasonal variation, and clinical presentations of recorded cases of cryptococcal, tuberculous, bacterial, and viral meningitis. We envision that understanding the epidemiology of meningitis will help focus public health resources for prevention, early diagnosis and meningitis treatment. More so, the Free State had the fourth highest incidence of meningococcal disease in 2017 [8].

## Methods

An audit of all adult meningitis cases, managed at the National District Hospital (NDH) Bloemfontein, Free State Province, South Africa between January 2017 and December 2019 was done during this retrospective, observational study.

**Study setting:** the NDH Bloemfontein is a government-funded district hospital in the Mangaung Metropolitan Municipality and part of the academic training facilities in Bloemfontein Free State province. The hospital consists of 197 inpatient beds that serves the greater Bloemfontein area, a community of about 567,000. The NDH serves as a referral centre to 2 community health centres and 15 primary healthcare clinics. The NDH is a level one hospital with a stepdown facility.

**Sampling:** a convenience sampling method was adopted for this study since all suitable cases of meningitis managed at the NDH during the 36-month period (January 2017 to December 2019) were included in this study. The following inclusion and exclusion criteria were utilized in this study.

**Inclusion criteria:** all cases of clinically diagnosed meningitis combined with laboratory evidence of meningitis subtypes managed at the NDH Bloemfontein between 1^st^ January 2017 and 31^st^ December 2019. Cases in patients aged ≥ 13 years were included in the study. This is because patients aged 13 years are often admitted and managed in the adult wards in the NDH Bloemfontein, as with most state hospitals in South Africa.

**Exclusion criteria:** cases not meeting the inclusion criteria were excluded.

**Data source:** particulars (name and hospital number) of patients (≥ 13 years) that were managed for meningitis during the 36-month period of study were identified from the adult ward patient register. The ward patient register is a paper-based log of daily ward/hospital admissions. Information such as patient demographics (age, gender, ethnicity etc.) and diagnosis-on-admission for each patient admitted are captured on the register. This is standard practice in most state hospitals in our setting. A list of patients diagnosed and treated for meningitis was made using the particulars obtained from the ward patient register. The generated list was then submitted to the hospital records department for the manual retrieval of each case files.

**Data management and processing:** all retrieved files were reviewed for appropriateness and case with significantly missing information were excluded from the study. Data were captured using a data collection sheet designed for the purpose of this study, based on trends observed in similar studies reviewed [6]. The data collection sheet was pre-tested on the first ten files (in succession). This ensured that the variables on the data sheet are well and correctly structured. It also ensured that every aspect was covered and that the aim of this study was attained. No significant changes resulted from the pre-testing, and the data obtained from the ten patient files were included in overall data of the study. The data collection sheet for each case file was filled in by one of the researchers on the grounds of the NDH in an office next to the hospital records department. Data captured include demographic details (i.e. gender, age group, ethnic group and residential area); information pertaining to meningitis (i.e. the time (year) of presentation); clinical state on presentation (i.e. symptoms, signs, vital signs, the Glasgow Coma Scale, medical co-morbidities); the distribution of meningitis subtypes (i.e. bacterial, tuberculous, viral and cryptococcal); and the outcome of these cases (i.e. discharged with or without any complication, transferred to higher level of care, or death). Data was entered into an Excel spreadsheet (Microsoft Corp, Redmond, WA, USA) for review and analysis.

**Data analysis:** data was analysed using R version 4.0.2 (R Foundation for Statistical Computing, Vienna, Austria). For continuous data, the results were summarised into mean and standard deviation for symmetric variables and median and inter-quartile range for asymmetric variables. Categorical data were summarised into frequencies and percentages and presented as frequency tables or histogram. Chi-squared test and Cramér's V were used to examine the existence and the strength of an association between cross-tabulated variables.

**Ethics:** approval to conduct the study was granted by the Health Sciences Research and Ethics Committee (HSREC) of the University of the Free State (UFS-HSD2020/0419/2909). Permission to make use of patient documents from the NDH was obtained from the Free State Department of Health. The data retrieved from the patients’ case files for the purpose of this study were handled confidentially and no identifying information was captured on the data sheet.

## Results

Of the 540 registered cases of meningitis, only 236 case files had sufficient information and were included in the study. The remaining 304 case files either couldn´t be retrieved or had incomplete information.

**Figure 1:**
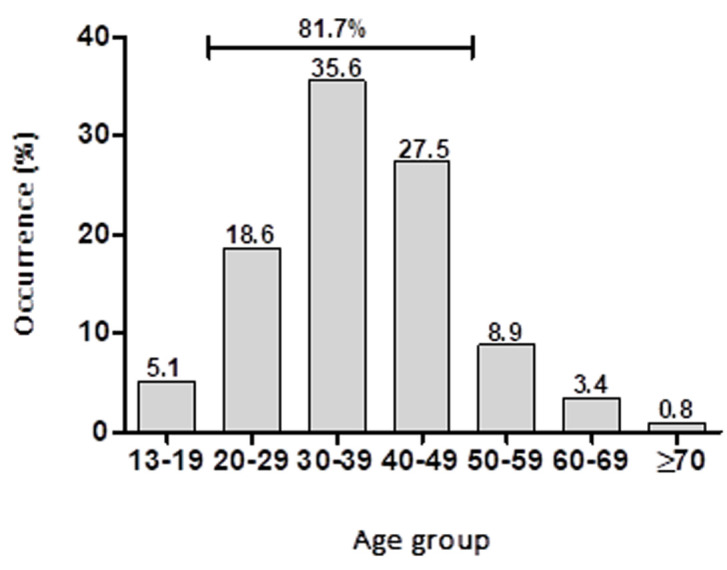
occurrence of meningitis amongst age groups

**Socio-demographic characteristics profile:** the majority (93.2%; n = 220) of the patients managed for meningitis were black, white made 1.7% (n = 4), coloured 3.4% (n = 8), and Asian 0.4% (n =1). Three patients had missing ethnicity. The majority (92.4%; n = 218) lived in the rural area. The mean age (SD) of the patients was 37.5 years (SD = 11.38) with the majority (55.5%; n = 131) being males. The male:female ratio was 1.25:1. The majority (35.6%; n = 84) of patients were in the age group 30-39 years (Figure 1). There was a high incidence of meningitis 81.7% among patients with ages 20 to 49 years.

**Meningitis sub-types:** tuberculous (37.3%) and bacterial (36%) were the most prevalent forms of meningitis followed by cryptococcal (16.1%) and viral (10.6%) meningitis.

**Figure 2:**
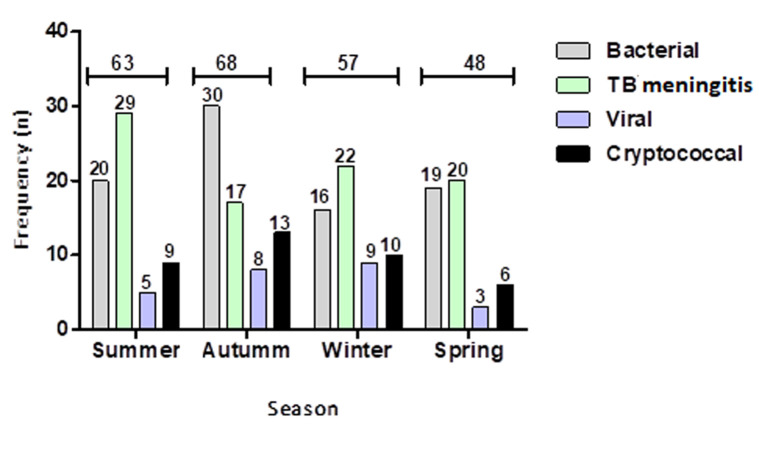
seasonal variation in the occurrence of meningitis in national district hospital Bloemfontein between 2017 and 2019

**Seasonal occurrence:** the majority of patients presented during autumn (28.8%; n = 68) with mostly bacterial meningitis infections (44.1%) (χ^2^=24.2; Cramér´s V = 0.2; p = 0.8) (Figure 2). Tuberculosis meningitis accounted for the majority of cases seen in summer and winter (46% and 38.6%, respectively). Bacterial and TB meningitis had similar occurrence in spring and accounted for 81.3% of cases seen during this period.

**Figure 3:**
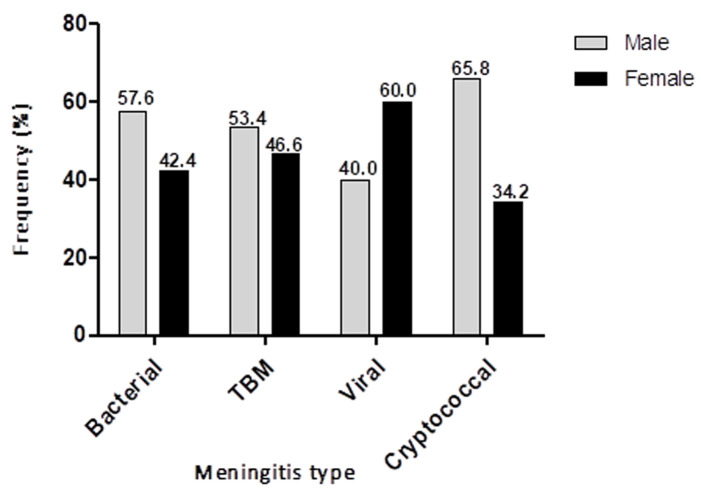
gender variation in the occurrence of meningitis in national district hospital Bloemfontein between 2017 and 2019

**Gender and occurrence of meningitis:** with the exception of viral meningitis, there was a higher prevalence of bacterial, TB and cryptococcal meningitis in the males than females [57.6% vs 42.4%; 53.4% vs 46.6%; 40% vs 60%; 65.8% vs 34.2%, respectively] (χ^2^=4.3; Cramér's V = 0.1; p = 0.2) (Figure 3).

**Figure 4:**
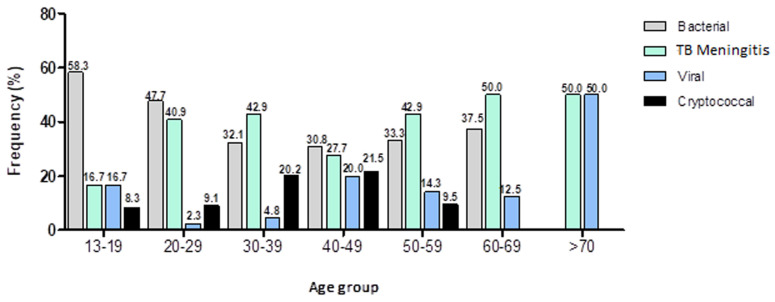
occurrence of meningitis types across age groups

**Age group and meningitis sub-types:** bacterial meningitis cases were reported in the 13-19, 20-29, and 40-49 age groups (58.3%, 47.70%, and 30.8%), while the TBM cases in age groups 30-39, 50-59, and 60-69 (42.9%, 42.9%, and 50%). Cryptococcal meningitis was mostly prevalent in all age groups except for 60-69 and ≥ 70 years (χ^2^=30.1; Cramér´s V = 0.2; p = 0.02) (Figure 4).

**Figure 5:**
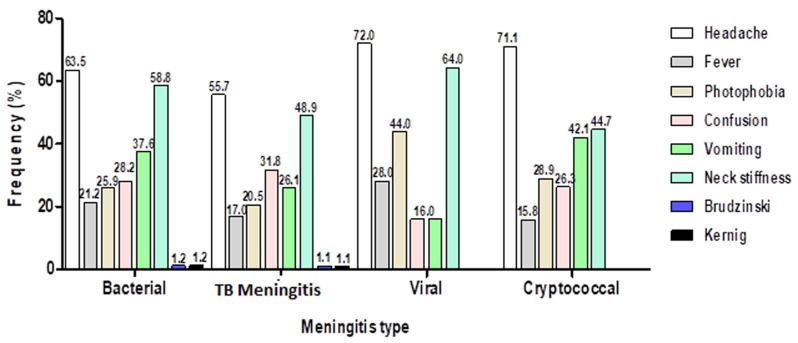
frequency of symptoms and signs in meningitis type

**Symptoms and signs on presentation:** Table 1 shows that headache (n = 148; 62.7%) and neck stiffness (n =126; 53.4%) were the two most prevalent symptoms, while nausea (n = 20; 8.5%) and convulsion (n = 13; 5.5%) were the least common symptoms. Only 2 patients (0.85%) had Kernig’s and Brudzinski’s signs reported in their case files. Further analysis as shown in Figure 5 reveals that the three most common symptoms in the different meningitis types are as follows: bacterial meningitis - headache (63.5%), neck stiffness (58.8%) and vomiting (37.6%); TB meningitis (TBM) - headache (57.7%), neck stiffness (48.9%) and confusion (31.8%); viral meningitis - headache (72.0%), neck fitness (64.0%) and photophobia (44.0%); cryptococcal meningitis - headache (71.1%), neck stiffness (44.7%) and vomiting (42.1%)

**Table 1:** frequency of symptoms and signs on presentation

Clinical feature	n (%)
Headache	148 (62.7)
Neck stiffness	126 (53.4)
Vomiting	75 (31.8)
Confusion	66 (28)
Photophobia	62 (26.3)
Fever	46 (19.5)
Body weakness	42 (17.8)
Nausea	20 (8.5)
Convulsion	13 (5.5)
Kernig	2 (0.85)
Brudzinski	2 (0.85)

**Glasgow Coma Scale (GCS):** the majority (n = 153; 64.8%) of patients had normal GCS, 74 (31.4%) had GCS between 9 and 14, while 3 (1.3%) had GCS ≤8. Glasgow coma scale was not recorded for six patients.

**Co-morbidities:** the two major co-morbidities were HIV (n = 197; 83%) and hypertension (n = 62; 26.3%). The majority (n = 78; 39.6%) of HIV cases was seen in patients with TB meningitis, while 33.5% (n = 66) bacterial, cryptococcal (n = 37; 18.8%) and viral meningitis (n = 16; 8.1%). Of the 197 patients with HIV co-infection, 163 (82.7%) were on antiretroviral therapy (ART), and 76 (38.6%) had CD4 count <100 cells/mm^3^.

**Outcomes:** the majority (n = 197; 83.5%) of the patients were discharged (after treatment course) without complications, seventeen (7.2%) were transferred to tertiary healthcare facilities for more specialized care, while 22 (9.3%) patients died. Of the 22 patients who died, 63.6% (n = 14) were males, in the age group 40-49 (n = 7; 31.8%), with TB meningitis (n = 12; 54.5%), were HIV positive (n = 20; 90.9%), had CD4 cell count <100 cells/mm^3^ (5%), were on ART (n = 14; 63.6%), and had GCS of 9-14 (n = 13; 59.0%). Of all the meningitis sub-types, TBM had the highest fatality rate (Table 2).

**Table 2:** fatality rates of meningitis sub-types

Meningitis sub-types	Outcome			Total cases (n)	Fatality rate (%)
	Discharged without complication (n)	Referred (n)	Died (n)		
Bacterial	71	7	7	85	8.2
TBM	70	6	12	88	13.6
Viral	22	3	0	25	0
Cryptococcal	34	1	3	38	7.9

## Discussion

Findings by this present study suggests that the risk of contracting meningitis in Bloemfontein may be greater in the rural than in the urban area. This findings is contrary to report from similar study in Tennessee, United States, where it was reported that the risk of contracting meningitis is greater in the urban than in the rural area [9]. The huge disparity between urban and rural cases in the current study is possibly attributed to the fact that the majority of people who utilize the public/state hospitals are from poor rural community because they often lack medical insurance. It has been reported that factors such as poverty, absence of adequate infrastructure, lack of access to health services, and degraded living environments makes rural dwellers in Africa susceptible to communicable diseases [10]. It is probable that these factors may account for the higher occurrence of meningitis in rural communities in Bloemfontein. In addition, the fact that the majority of those who live in the poorer rural areas in South Africa and indeed Free State province are black Africans may account for the higher occurrence of meningitis amongst black Africans as seen in this study. Findings by this present study that shows a higher prevalence of meningitis in Males more than Females corroborates findings by Meiring *et al*. (2019) [8]. Our findings, which shows that the highest number of recorded meningitis cases was found within the age range of 20-49 years is consistent with similar findings by Yerramilli *et al*. (2017) [11]. This suggest that individuals aged 20-49 years are possibly at higher risk of developing meningitis compared to other age groups.

The high prevalence of HIV infection and tuberculosis (TB) in SA has led to changes in the spectrum of causative agents implicated in meningitis among adults [6]. According to a 2018 global AIDS monitoring report, the Free State province had the second highest HIV prevalence among adults aged 15-49 years, of all the nine provinces [12]. *Mycobacterium tuberculosis* and *Cryptococcus neoformans* are some of the common pathogens implicated in meningitis among adults in high HIV prevalence settings [13]. Our findings show that TB and cryptococcal meningitis accounted for more than half (53.2%) of the meningitis cases that presented at the National District Hospital during the study period. This corroborates the findings by Thinyane *et al*. (2015), where it was reported that TB meningitis was the most frequent diagnosis (39%), in a study aimed at investigating the clinical presentation, aetiology, and outcomes of meningitis among adult patients in a hospital in Maseru, Lesotho, Southern Africa [14]. More so, we found that the majority (n = 197; 83%) of the patient had HIV as a co-morbidity. It is very likely that the high burden of HIV infection in the province and in the Bloemfontein area is responsible for the high incidence of TB and cryptococcal meningitis reported herein. While there is no significant difference in the seasonal occurrence of the disease, we found that the majority (n = 68; 28.8%) of cases occurred in autumn, with bacterial meningitis responsible for most of the cases managed during this period. This is contrary to prior findings that reported a surge in bacterial meningitis (*Neisseria meningitidis*) during the winter and spring months [8].

Prior studies have reported on the seasonal variation in the incidence of tuberculosis, with peak incidence recorded during summer or spring [15,16]. This could account for the majority of TBM cases recorded in summer in the present study. Furthermore, the high transmission rates of tuberculosis reported in the winter season [15] could be responsible for high occurrence of TBM during the winter season. Spending more time indoors and overcrowding, increased humidity, diminished amounts of natural ultraviolet light and low airflow has been suggested to provide a suitable environment for *M. tuberculosis* transmission during winter [15]. It is very likely that these factors could also cause an upsurge in the transmission of TBM during the winter season. It has been reported that susceptibility to viral infections as well as their severity are higher in men than in women [17]. Similarly, several studies have reported on the high incidence of viral meningitis in males more than females [18,19]. This is contrary to our findings that shows a higher incidence of viral meningitis in females compared to males (Figure 3). Primary HIV infection is an important cause of aseptic (viral) meningitis and in South Africa, the prevalence of HIV infection is disproportionately higher in females than in males [12,20]. This may account for the increased rate of viral meningitis in females. Furthermore, Herpes simplex virus (HSV-2) meningitis, a complication of primary genital herpes has been reported to occur more frequently in females [21]. It is likely that the cases of viral meningitis reported in this present study are because of HSV-2 infection.

The triad of fever, headache, and neck stiffness has been tagged the classic symptoms of meningitis [11,22]. Similarly, in this present study, we found that headache (62.7%) and neck stiffness (53.4%) were the leading presenting symptoms. In addition, our findings reveal that symptoms and signs may vary according to the meningitis sub-type (Figure 5). HIV was the most common co-morbidity found amongst the patients (n = 197; 83%). This mimics the endemicity of the disease in South Africa and indeed the Free State province and corroborate findings by Thinyane and colleagues [14]. Tuberculosis meningitis (TBM) is associated with high rates of death and severe neurological disability [23]. Similarly, findings in the present study show that fatality was higher among patients with TBM. In a study aimed at investigating the mortality in hospitalized patients with TBM, Soria *et al*. (2019) reports that mortality was higher among male patients with HIV infections, aged over 40 years [24]. This corroborates our findings that show that fatality was higher among male patients who had TBM, aged 40-49 years, and were HIV positive. Further, we found that mortality was higher in patients with low CD4 cell count. Factors such as severe illness, low CD4 cell count, and presence of MDR-TB are associated with poor prognosis of TBM in HIV-infected patients [25,26]. It is plausible that these factors contribute to the high mortality seen in patient with TBM in this present study.

## Conclusion

Our study suggests that combining information on patient demography, co-morbidities, clinical presentation, and examination findings can substantially contribute to raising clinical suspicion, leading to swift identification, diagnosis, and treatment of patients. Adequate documentation and proper record keeping should be continuously encouraged in order to ensure that clinical records can be accessed when required. There is need for a more comprehensive study that will include cases managed at private hospitals and other surrounding state hospitals in order to obtain a more generalizable epidemiological profile of meningitis in Bloemfontein.

**Limitations:** our study is limited in part by poor record keeping, as some of the ward registers were either unavailable or missing. Our study findings are generalizable only to patients who presented at the NDH or similar hospital settings, and not those who receive care at private hospitals and other state hospitals in Bloemfontein that serve as referral centers for suspected cases of meningitis. This suggests that our data may not accurately describe the true epidemiological profile of meningitis in the whole of Bloemfontein. Finally, the impact of the ongoing COVID-19 pandemic cannot be overlooked as some wards were used as a PUI (person under investigation) for COVID-19 ward and this made it impossible for the researchers to gain access to the registers in those wards.

### What is known about this topic

- Meningitis remains a debilitating illness of serious public health concern, globally;
- The annual incidence rate of meningitis in South Africa is estimated to be 4 per 100 000 cases;
- High dual burden of HIV infection and tuberculosis (TB) has altered the spectrum of causes and outcome of meningitis among adults in SA.

### What this study adds

- The pattern of seasonal occurrence of meningitis sub-types in adult patients in Bloemfontein, South Africa;
- Fatality rates of meningitis sub-types in a HIV and TB endemic community, Bloemfontein, South Africa;
- Frequency of meningitis sub-types among adult population in Bloemfontein, South Africa.
